# A dual gene-specific mutator system installs all transition mutations at similar frequencies *in vivo*

**DOI:** 10.1093/nar/gkad266

**Published:** 2023-04-18

**Authors:** Daeje Seo, Bonghyun Koh, Ga-eul Eom, Hye Won Kim, Seokhee Kim

**Affiliations:** Department of Chemistry, Seoul National University, 1 Gwanak-ro, Gwanak-gu, Seoul 08826, Republic of Korea; Department of Chemistry, Seoul National University, 1 Gwanak-ro, Gwanak-gu, Seoul 08826, Republic of Korea; Department of Chemistry, Seoul National University, 1 Gwanak-ro, Gwanak-gu, Seoul 08826, Republic of Korea; Department of Chemistry, Seoul National University, 1 Gwanak-ro, Gwanak-gu, Seoul 08826, Republic of Korea; Department of Chemistry, Seoul National University, 1 Gwanak-ro, Gwanak-gu, Seoul 08826, Republic of Korea

## Abstract

Targeted *in vivo* hypermutation accelerates directed evolution of proteins through concurrent DNA diversification and selection. Although systems employing a fusion protein of a nucleobase deaminase and T7 RNA polymerase present gene-specific targeting, their mutational spectra have been limited to exclusive or dominant C:G→T:A mutations. Here we describe eMutaT7^transition^, a new gene-specific hypermutation system, that installs all transition mutations (C:G→T:A and A:T→G:C) at comparable frequencies. By using two mutator proteins in which two efficient deaminases, PmCDA1 and TadA-8e, are separately fused to T7 RNA polymerase, we obtained similar numbers of C:G→T:A and A:T→G:C substitutions at a sufficiently high frequency (∼6.7 substitutions in 1.3 kb gene during 80-h *in vivo* mutagenesis). Through eMutaT7^transition^-mediated TEM-1 evolution for antibiotic resistance, we generated many mutations found in clinical isolates. Overall, with a high mutation frequency and wider mutational spectrum, eMutaT7^transition^ is a potential first-line method for gene-specific *in vivo* hypermutation.

## Graphical Abstract

**Graphical Abstract:**
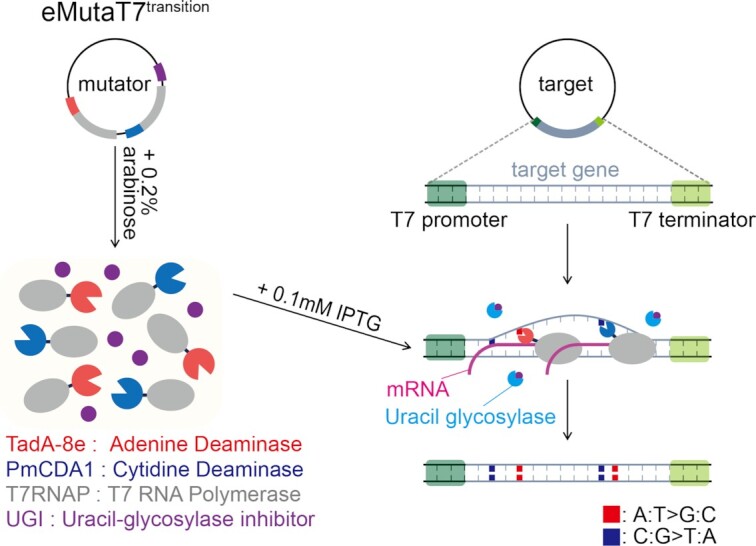
eMutaT7^transition^ uses two chimeric mutators, eMutaT7^PmCDA1^ (PmCDA1_T7RNAP) and eMutaT7^TadA-8e^ (TadA-8e_T7RNAP), and co-expresses a uracil-glycosylase inhibitor (UGI). When T7 RNA polymerase transcribes the target gene between T7 promoter and T7 terminator, deaminase enzymes introduce C:G → T:A (cytidine deaminase) and A:T → G:C (adenine deaminase) mutations.

## INTRODUCTION

Directed evolution is a powerful approach that mimics natural evolution to improve biomolecular activity (1,2). Traditional directed evolution relies on *in vitro* gene diversification such as error-prone PCR or randomized oligonucleotide pools (2). In contrast, continuous directed evolution (CDE) adopts *in vivo* hypermutation, allowing for simultaneous gene diversification, selection, and replication in cells; this technique significantly enhances the depth and scale of biomolecular evolution (3–5). As random mutagenesis in the genome is highly deleterious to cells, *in vivo* hypermutation methods should aim to introduce mutations in a relatively narrow region around the target gene (5).

Various methods for targeted *in vivo* hypermutation have been reported recently, based on distinct molecular principles and thus presenting assorted target ranges (3–5). Several CRISPR-Cas-based methods (e.g. EvolvR (6), CRISPR-X (7), base editors (8–11), and prime editors (12)) install mutations in smaller regions of a gene. OrthoRep (13) uses an orthogonal error-prone DNA polymerase and generates mutations on a plasmid. Virus-based methods, such as PACE (14) and mammalian cell-based systems (15,16), mutate the entire viral genome. Considering that most directed evolution experiments focus on a single protein, the ideal target range is a single gene of interest. The gene-specific targeting was achieved through homologous platforms that use chimeric mutator proteins, generated by fusing a nucleobase deaminase to an orthogonal T7 RNA polymerase (T7RNAP) (17–22).

The deaminase-T7RNAP system was first reported in bacteria (MutaT7) (17) and further extended to mammalian cells (TRACE) (18), yeast (TRIDENT) (21) and plants (22). We previously demonstrated that the mutation frequency of MutaT7 could be enhanced 7- to 20-fold with a more efficient cytidine deaminase, *Petromyzon marinus* cytidine deaminase (PmCDA1) (20). This PmCDA1_T7RNAP mutator (previously termed eMutaT7, but here renamed eMutaT7^PmCDA1^) generated ∼4 mutations per 1 kb per day in *Escherichia coli*, representing the fastest gene-specific *in vivo* mutagenesis. The major limitation of eMutaT7^PmCDA1^ is a narrow mutational spectrum: it mainly generates C→T mutations on the coding strand and, with the Shoulders group's dual promoter/terminator approach that induces transcription in both directions, introduces C→T and G→A mutations (C:G→T:A) (17,20). Mutations could be expanded to A→G and T→C mutations (A:T→G:C) with engineered tRNA adenosine deaminases, TadA-7.10 (11,19) and yeTadA1.0 (21), but they either had a mutation frequency much lower than eMutaT7^PmCDA1^ (19), or presented C:G→T:A as dominant mutations (∼95%) in nonselective conditions when combined with PmCDA1_T7RNAP (21).

Here, we report on eMutaT7^transition^, a new dual mutator system that introduces all transition mutations (C:G→T:A and A:T→G:C) at comparable frequencies. The eMutaT7^transition^ system uses two mutators, eMutaT7^PmCDA1^ and eMutaT7^TadA-8e^. The latter is the fusion of T7RNAP and a recently evolved *E. coli* adenosine deaminase, TadA-8e (23), which had much higher mutational activity than the previously evolved TadA-7.10 (11). We optimized the expression of the two mutators and a uracil glycosylase inhibitor, and demonstrated that the frequencies of the C:G→T:A and A:T→G:C mutations were not significantly different. Furthermore, overall mutation frequency was not markedly reduced. eMutaT7^transition^ also promoted rapid continuous directed evolution of antibiotic resistance with various transition substitutions, suggesting that it is a viable alternative for gene-specific *in vivo* hypermutation with an improved mutational spectrum.

## MATERIALS AND METHODS

### Materials

All PCR experiments were conducted with KOD Plus neo DNA polymerase (Toyobo, Japan). T4 polynucleotide kinase and T4 DNA ligases were purchased from Enzynomics (South Korea). Plasmids and DNA fragments were purified with LaboPass^TM^ plasmid DNA purification kit mini and LaboPass™ Gel extraction kit (Cosmogenetech, South Korea). Sequences of all DNA constructs in this study were confirmed by Sanger sequencing (Macrogen, South Korea and Bionics, South Korea). Antibiotics (carbenicillin, chloramphenicol, kanamycin), arabinose, and Isopropyl β-d-1-thiogalactopyranoside (IPTG) were purchased from LPS solution (South Korea). Streptomycin was purchased from Sigma Aldrich. Tetracycline was purchased from Bio Basic. Cefotaxime and ceftazidime were purchased from Tokyo chemical industry (Japan). H-*p*-Chloro-dl-Phe-OH (*p*-Cl-Phe) was purchased from Bachem (Switzerland).

### Plasmid and *E. coli* strain construction

*Escherichia coli* strains, plasmids, and primers used in this study are listed in Supplementary Tables S1-3, respectively. Genes for adenine deaminase TadA-7.10 (11) and TadA-8e (23) were synthesized from Gene Universal (USA). Genes for the *Petromyzon marinus* cytidine deaminase (PmCDA1), XTEN linker and T7 RNA polymerase (T7RNAP) were amplified from the plasmid expressing eMutaT7 (pHyo094) (20). Genes for adenine deaminases (TadA-7.10 or TadA-8e), linker, and T7RNAP were linked by *in vivo* assembly (IVA) cloning (24). Plasmids expressing PmCDA1_XTEN_T7RNAP (eMutaT7^PmCDA1^), TadA-8e_XTEN_T7RNAP (eMutaT7^TadA-8e^) and UGI were cloned by IVA cloning. Also, genes encoding triply-fused proteins, UGI_PmCDA1_T7RNAP, PmCDA1_TadA-8e_T7RNAP, and TadA-8e_PmCDA1_T7RNAP, were also constructed by IVA cloning. All target plasmids (pDae117, *malE* (maltose binding protein); pDae118, *gfp* (green fluorescent protein); pDae119, *malE* and *gfp*) were constructed in a low copy-number plasmid (pHyo182) by IVA cloning.

All plasmids expressing variants of mutators or targets (mutation, deletion, and insertion) were constructed using the site-directed mutagenesis PCR method (25). Plasmids expressing eMutaT7^PmCDA1^ and UGI in different conditions (deletion of UGI, an optimized ribosomal binding site (RBS) for UGI, or a constitutive promoter for UGI) were made on pHyo094. Sequence of the optimized RBS region is AACAGAGCGCGCTCTGTTTGAGTACTAGCAATAAATAAGGAGGATTTTTT (the underlined sequence indicates RBS) (26). Plasmids harboring TadA-8e were made on pDae029. Plasmids expressing PmCDA1_TadA-8e_T7RNAP with different linkers were constructed on pDae036.

For evolution of antibiotic resistance, a target plasmid (pGE158) was constructed from pHyo245, which contains the *pheS*_A294G gene between dual promoter/terminator pairs in a low-copy-number plasmid (20): Ampicillin resistance gene in pHyo245 was replaced with tetracycline resistance gene and *pheS*_A294G was replaced with the *TEM-1* gene by IVA cloning. Tetracycline resistance gene was amplified from the plasmid pREMCM3 (27) and the *TEM-1* gene was obtained from pHyo182 (20).

W3110 *ΔalkA Δnfi* strain (cDJ085) and W3110 *ΔlacZ::KanR-P_T7_-gfp-T_T7_* (cDJ092) were constructed by homologous recombination method (28) The *alkA* and *nfi* genes in W3110 were replaced with the streptomycin resistance gene and the kanamycin resistance gene, respectively. The *lacZ* gene in W3110 was replaced with the kanamycin resistance gene and *gfp* gene. 30 μg/ml of streptomycin or kanamycin was used for selection. Proper gene deletion was confirmed by colony PCR using 2X TOP simple^TM^ DyeMIX-Tenuto (Enzynomics).

### *In vivo* hypermutation

Three biological replicates of W3110 or the *Δung* strain (cHYO057) harboring a mutator plasmid and a target plasmid (pHyo182, pDae117, pDae118, and pDae119 for a single promoter) were grown overnight in LB medium with 35 μg/ml chloramphenicol and 50 μg/ml carbenicillin (cycle #0). On the following day, the overnight cultures were diluted 100-fold in a fresh LB medium supplemented with 35 μg/ml chloramphenicol, 50 μg/ml carbenicillin, 0.2% arabinose, and 0.1 mM IPTG in a 96-deep well plate (Bioneer, South Korea) and incubated at 37°C with shaking (cycle #1). Bacterial cells were diluted every 4 hours and this growth cycle was repeated up to 20 times for accumulation of mutations. At the end of cycle, a fraction of cells were stored at –80°C with 15% glycerol. To identify mutations in the target gene, cells at cycle #20 were streaked on LB-Agar plates with 35 μg/ml chloramphenicol and 50 μg/ml carbenicillin. Three or six colonies were randomly chosen for isolation of target plasmids. The target genes in the purified target plasmids were sequenced by Sanger sequencing. Mutations were counted in the region between 147-bp upstream and 138-bp downstream of the *pheS*_A294G gene (total 1269 bp), *malE* gene (total 1389 bp), and *gfp* gene (total 1005 bp). Primer 314 and 315 were used for amplification and sequencing of the target gene that has a single promoter system.

### PheS_A294G suppression assay

Suppression frequency of the *pheS*_A294G toxicity was determined as previously described (20). Cells obtained at the endpoint of each cycle (overnight culture for cycle #0) were diluted to OD_600_ ∼0.2. Serial 10-fold dilutions of cells (5 μl) using LB broth were placed on YEG-agar plates with or without additives (16 mM *p*-Cl-Phe, 0.2% arabinose, and 0.1 mM IPTG) and grown overnight at 37°C. On the following day, the number of colonies on each condition was counted to calculate the suppression frequency. The suppression frequency was calculated as N_1_/N_0_ (N_1_: colony forming unit (CFU) in the *p*-Cl-Phe plates and N_0_: CFU in plates without *p*-Cl-Phe).

### Assays for cell viability and off-target mutagenesis

Cell viability and off-target mutagenesis were assayed as previously described (20). Overnight cultures of the cells harboring the plasmid expressing eMutaT7^TadA-8e^, no mutator, or MP6 were diluted 100-fold in LB supplemented with 35 μg/ml chloramphenicol and grown to a log phase (OD_600_ = 0.2–0.5) at 37°C. Cells were diluted to OD_600_ ∼0.2 and serial 10-fold dilutions of cells (5 μl) using LB broth were placed on LB-agar supplemented with 35 μg/ml chloramphenicol and 0.2% arabinose. After overnight growth at 37°C, the number of colonies on the plates were counted to calculate CFU/ml. To evaluate the off-target mutagenesis via rifampicin resistance, cells taken at cycle #0 and cycle #20 were grown to log phase in LB supplemented with 35 μg/ml chloramphenicol and 50 μg/ml carbenicillin, and subjected to viability assay on plates with or without rifampicin (50 μg/ml).

### Fluctuation analysis

Fluctuation analysis was performed as previously described (29). Cells harboring a mutator plasmid (pDae079, eMutaT7^transition^) and a target plasmid (pHyo182 for a T7 promoter; pDae120 for constitutive promoter (BBa_J23100)) were grown overnight in LB medium with 35 μg/ml chloramphenicol and 50 μg/ml carbenicillin. The cultures were diluted 1:10^6^ with induction media containing 35 μg/ml chloramphenicol, 50 μg/ml carbenicillin, 0.2% arabinose and 0.1 mM IPTG, and divided into 32 wells (50 μL each) in a 96-deep well plate. This plate was sealed and incubated for 6 hours (pHyo182) or 16 hours (pDae120) at 37°C with shaking. To assess the total cell counts, 8 cultures were resuspended and plated on a YEG-agar plate at the required dilutions. The remaining 24 cultures were resuspended using a pipette, and placed on YEG-agar plates with additional ingredients (16 mM *p*-Cl-Phe, 0.2% arabinose, and 0.1 mM IPTG). Colonies on YEG-agar plates with or without additives were counted after an overnight incubation.

The Ma-Sandri-Sarkar (MSS) maximum likelihood method was used to compute the loss-of-function mutation rate (30), and the 95% confidence intervals (CIs) were calculated as previously described (29). The FALCOR webtool (https://lianglab.brocku.ca/FALCOR) was used with both of these methods (31). The calculated loss-of-function mutation rates serve simply as comparative estimates for per-base-pair mutation rates in our study.

### High-throughput sequencing and data analysis

Cells taken at cycle 0 and cycle 20 were sequenced as previously described (20). Cells taken at cycle 0 (*n* = 1) and cycle 20 (*n* = 3) were grown in 15 ml of LB broth without arabinose and IPTG, and the plasmids were extracted with Plasmid DNA Miniprep Kit. The 3288 bp DNA fragments containing the *pheS*_A294G gene were amplified using primer 512 and 513 covering from 999 bp upstream from T7 promoter and to 1020 bp downstream from T7 terminator. The 2 × 151 paired-end sequencing library was constructed using TruSeq Nano DNA Kit and were sequenced using NovaseqTM (Illumina; operated by Macrogen).

The quality of the sequencing data was checked with FastQC (v0.11.8). Raw reads were trimmed to remove adapter sequences and low-quality end sequences using Trimmomatic (v0.38) (32). Processed data were aligned to the reference sequence (3288 bp) using Burrows-Wheeler Aligner (BWA v0.7.17) with MEM mode and BAM files generated by mapping were sorted using SAMtools (v1.9) (33,34). Sorted BAM files were subject to SAMtools mpileup to obtain a pileup output with maximum depth option, which was set as total number of trimmed reads, and output tag list option consisting of DP, DP4 and AD. Alleles for each locus were called using BCFtools (v1.9), which was a set of utilities of SAMtools package, with multiallelic-caller option. Allele count for each allele and ratio (each allele count/total allele count) were calculated based on AD information of VCF files.

### Statistical analysis

**Figure 1.**
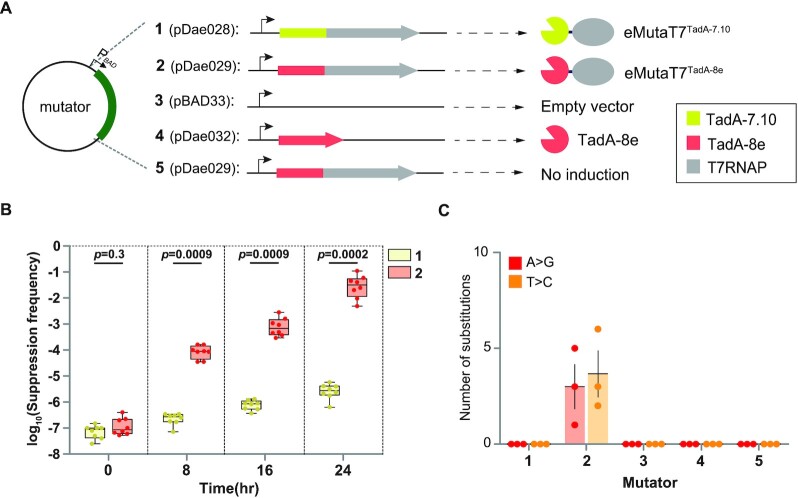
eMutaT7^TadA-8e^ rapidly introduces A→G and T→C mutations in the target gene. (**A**) Scheme of the tested mutators and conditions. (**B**) Frequency of the *pheS*_A294G toxicity suppression at each mutagenesis cycle for cells expressing eMutaT7^TadA-7.10^ (**1**) or eMutaT7^TadA-8e^ (**2**). Box limits indicate interquartile range; whiskers, minimum to maximum; center line, median; dots, individual data points (*n* = 8). *P* values were obtained with two-sided Mann–Whitney tests; *P* < 0.05 was considered significant. (**C**) Number of A→G (red) or T→C (orange) substitutions found in three clones obtained after 20 mutagenesis cycles. Numeric labels in the x-axis (**1**–**5**) indicate five different setups of mutagenesis shown in (A). Data are presented as dot plots with mean ± standard deviation (SD) (*n* = 3).

**Figure 2.**
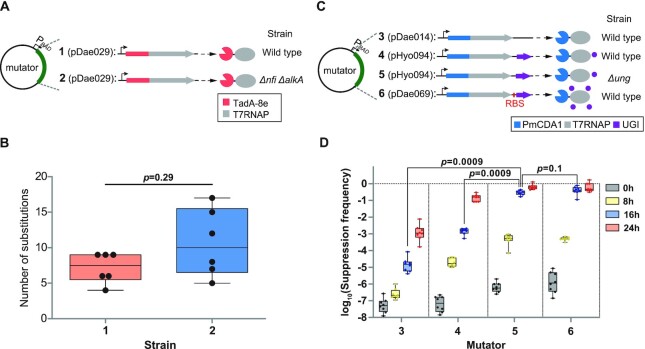
Optimization of eMutaT7^TadA-8e^- or eMutaT7^PmCDA1^-mediated *in vivo* hypermutation. (**A**) eMutaT7^TadA-8e^ activity was tested in wild-type (**1**) or *Δnfi ΔalkA* (**2**) strains. (**B**) Number of substitutions found in six clones from the two samples shown in (A) after 20 mutagenesis cycles. (**C**) eMutaT7^PmCDA1^ activity was tested without or with an optimized ribosomal binding site for *ugi* in wild-type or *Δung* strains. (**D**) Suppression frequency of the *pheS*_A294G toxicity at each mutagenesis cycle for cells evolved under the four different setups shown in (C) (**3**–**6**). In (B) and (D), box limits indicate interquartile range; whiskers, minimum to maximum; center line, median; dots, individual data points (*n* = 6 in (B) and *n* = 8 in (D)). *P* values were obtained with two-sided Mann–Whitney tests; *P* < 0.05 was considered significant.

**Figure 3.**
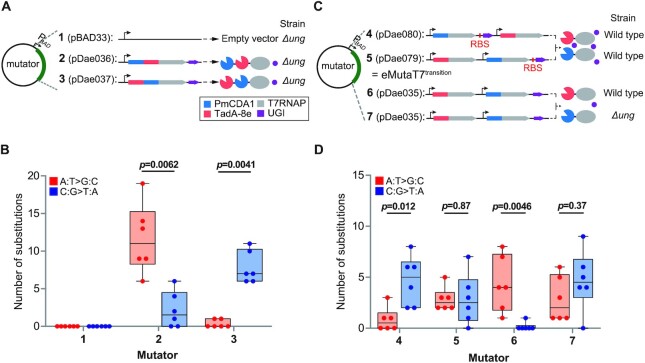
eMutaT7^transition^ rapidly introduces all transition mutations at similar frequencies. (**A**) Two triply-fused mutators were tested for equivalent incorporation of A:T→G:C and C:G→T:A substitutions. (**B**) Substitution counts in six clones from samples shown in (A) (**1**–**3**) after 20 mutagenesis cycles. (**C**) Four dual mutator systems were tested for equivalent incorporation of A:T→G:C and C:G→T:A substitutions. (**D**) Substitution counts in six clones from samples shown in (C) (**4**–**7**) after 20 mutagenesis cycles. In (B) and (D), box limits indicate interquartile range; whiskers, minimum to maximum; center line, median; dots, individual data points (*n* = 6). *P* values were obtained with two-sided Mann–Whitney tests; *P* < 0.05 was considered significant.

**Figure 4.**
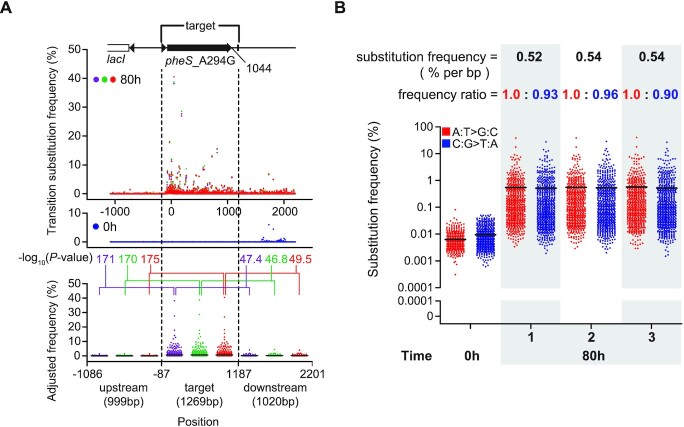
Illumina sequencing confirmed the rapid targeted *in vivo* hypermutation of eMutaT7^transition^ with similar frequencies of A:T→G:C and C:G→T:A substitutions. (**A**) Frequencies of all transition substitutions (top) in ∼3.3 kb DNA around the target gene at cycle 0 (*n* = 1; blue) or cycle 20 (*n* = 3; purple, green, and red), and collective substitution frequencies of the three regions of DNA (bottom; dot plots with averages shown as short black lines) at cycle 20 adjusted by subtracting frequencies at cycle 0. (**B**) Collective frequencies of A:T→G:C (red) and C:G→T:A (blue) substitutions in the target gene (dot plots with averages shown as short black lines). The ratios of A:T→G:C and C:G→T:A frequencies adjusted by subtracting frequencies at cycle 0 as well as the substitution frequencies were shown above. Y-axes above and below 0.0001% are in log- and linear-scale, respectively. *P* values were obtained with two-sided Mann–Whitney tests and presented as –log_10_(*P* value).

For high-throughput sequencing data (Figure 4 and Supplementary Figure S6), Mann-Whitney test (unpaired Wilcoxon test) was used to assess the significance of the substitution frequency caused by the eMutaT7^transition^ system. Calculation was conducted using Stata (USA). Statistical significance was determined with *P* values. *P* < 0.05 was considered significant for this experiment. For other data, statistical analyses comparing groups in pairs were performed using two-sided Mann–Whitney test (Figures 1–3, Supplementary Figures S1C, S3B, and S5B) without assuming that the data follow normal distribution or two-tailed Student's *t*-test (Supplementary Figure S1B) assuming that the data follow normal distribution. Calculation was conducted using GraphPad prism 5. *P* < 0.05 was considered significant.

### TEM-1 evolution and identification of the evolved mutants

TEM-1 evolution experiments were performed as previously described (20). Strains were grown in LB medium supplemented with 6 μg/ml tetracycline, 35 μg/ml chloramphenicol, 0.2% arabinose, and 0.1 mM IPTG. Cells were grown without selection pressure at the initial cycle. Then, multiple cultures were grown with different concentrations of an antibiotic (cefotaxime and ceftazidime) at the same time and the culture grown at the highest antibiotic concentration (OD_600_ > 1) were used for the next round of evolution. After the final cycle, the target plasmids were purified and re-inserted into fresh W3110 cells harboring the T7RNAP-expressing plasmid (pHyo183) for validation of antibiotic resistance. Twelve colonies were randomly selected for MIC measurement and those with high MIC values (five colonies with 400–1600 μg/ml MIC for CTX, three colonies with 4000 μg/ml MIC for CAZ) were subjected to the target gene sequencing by Sanger method.

### MIC determination

MIC values were measured as previously described (20). Cells were grown overnight in LB medium supplemented with 6 μg/ml tetracycline, 35 μg/ml chloramphenicol. They were diluted 10 000-fold into fresh LB broth with increasing concentrations of antibiotics (2-fold) in 96-deep well plates, and grown at 37°C with shaking (290 rpm) overnight. Final cell density (OD_600_) was measured by M200 microplate reader (TECAN, Switzerland).

## RESULTS AND DISCUSSION

### eMutaT7^TadA-8e^ promotes rapid gene-specific *in vivo* hypermutation

To date, TadA-8e is the most efficient TadA variant, presenting a rate constant (*k*_app_) 590 times higher than that of the previous TadA-7.10, and has been successfully used for genome editing (23). To evaluate their efficiency in gene-specific *in vivo* hypermutation, we fused TadA-7.10 and TadA-8e to the N-terminus of T7RNAP, creating eMutaT7^TadA-7.10^ and eMutaT7^TadA-8e^, respectively (Figures 1A, **1** and **2**). As in the previous characterization of eMutaT7^PmCDA1^ (20), we expressed the mutator and induced hypermutation in the target gene, *pheS*_A294G, which was inserted between T7 promoter and T7 terminator in a low-copy-number plasmid. We determined mutational suppression of the *pheS*_A294G toxicity by counting viable cells in the presence of *p*-chloro-phenylalanine (*p*-Cl-Phe), which is toxic to cells containing intact *pheS*_A294G. We performed 20 rounds of *in vivo* hypermutation (4 h growth and 100-fold dilution to a new medium for a single round) without *p*-Cl-Phe and then sampled cells at different time points for the cell viability assay. We found that the suppression frequencies of eMutaT7^TadA-8e^ were several orders of magnitude higher than the eMutaT7^TadA-7.10^ frequencies after 8 h, indicating that eMutaT7^TadA-8e^ induces gene-specific hypermutation much faster than eMutaT7^TadA-7.10^ (Figure 1B).

**Figure 5.**
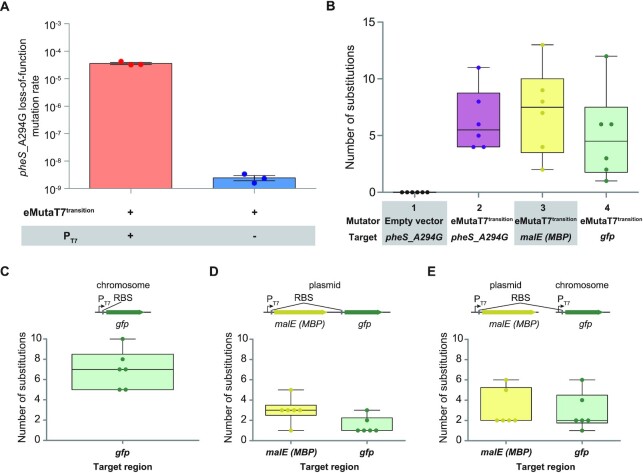
*In vivo* hypermutation activity and target tolerance of eMutaT7^transition^. (**A**) *pheS*_A294G loss-of-function mutation rates of eMutaT7^transition^ with or without T7 promoter, which is quantified using the FALCOR webtool. Data are presented as dot plots with mean ± standard deviation (SD) (*n* = 3). (**B**) Substitution counts in six clones from samples harboring a mutator (empty vector or eMutaT7^transition^) and a target plasmid (*pheS*_A294G, *malE* (maltose binding protein), or *gfp* (green fluorescent protein)). (**C**) Substitution counts in six clones from samples in which eMutaT7^transition^ targets the *gfp* gene in the chromosome (**D**) Substitution counts in six clones from samples in which eMutaT7^transition^ targets both the *malE* and *gfp* genes in the single target plasmid. (**E**) Substitution counts in six clones from samples in which eMutaT7^transition^ targets both the *malE* gene in the target plasmid and the *gfp* gene in the chromosome. In (B)–(E), box limits indicate interquartile range; whiskers, minimum to maximum; center line, median; dots, individual data points (*n* = 6).

To examine whether eMutaT7^TadA-8e^ generates mutations in the target gene, we randomly selected three clones from cells that had undergone 20 rounds of hypermutation and sequenced the target gene by Sanger method. We also included as negative controls cells that had an empty vector, expressed TadA-8e without T7RNAP, or contained the eMutaT7^TadA-8e^ plasmid without induction (Figures 1A, **3**–**5**). Notably, we found ∼6.7 substitutions per clone in the eMutaT7^TadA-8e^-expressing cells, while eMutaT7^TadA-7.10^-expressing cells and negative controls did not exhibit mutations (Figure 1C and Supplementary Figure S1A). This mutation frequency is definitely much higher than that of eMutaT7^TadA-7.10^ and only 2.4-fold lower than that of eMutaT7^PmCDA1^ (20). Interestingly, we identified nine A→G (45%) and eleven T→C (55%) mutations on the coding strand, indicating that eMutaT7^TadA-8e^ causes mutations on both DNA strands (Figure 1C and Supplementary Figure S1A). We observed that eMutaT7^TadA-8e^ neither noticeably reduced cell viability (Supplementary Figure S1B) nor induced rifampicin resistance (Supplementary Figure S1C). This result suggests that eMutaT7^TadA-8e^ does not generate significant off-target mutations in the genome.

### Deletion of genes associated with hypoxanthine repair does not significantly increase eMutaT7^TadA-8e^ activity

In the eMutaT7^PmCDA1^ system, deletion of a gene encoding a uracil-DNA glycosylase (UNG) enhanced the mutation frequency (20). UNG removes uracil (deaminated cytosine) and initiates the base excision repair pathway (35). Likewise, we hypothesized that the deletion of genes encoding hypoxanthine (deaminated adenine)-removing enzymes would further increase the eMutaT7^TadA-8e^-mediated mutation frequency. We prepared a strain in which two genes involved in hypoxanthine repair, *nfi* (36,37) and *alkA* (38), are deleted and analyzed eMutaT7^TadA-8e^-mediated hypermutation (Figure 2A). Twenty rounds of targeted hypermutation revealed that the mutation frequency in the *Δnfi ΔalkA* strain did not increase significantly from the wild-type level (11 and 7.2 substitutions per clone on average, respectively) (Figure 2B and Supplementary Figure S2). Because a DNA repair enzyme often reduces the mutation rate by more than an order of magnitude (39–42) and the construction of a gene deletion strain requires additional experimental steps, we concluded that the *Δnfi ΔalkA* strain has no obvious advantage over the wild-type strain for eMutaT7^TadA-8e^. No significant increase of mutations in the *Δnfi* strain was also previously observed (19).

### Optimized expression of uracil glycosylase inhibitor increases eMutaT7^PmCDA1^ activity

Although we co-expressed a UNG inhibitor (UGI) with eMutaT7^PmCDA1^ from the plasmid pHyo094, we did not obtain an efficiency level that matched the *Δung* strain (20). Proper UGI expression can greatly expand eMutaT7^PmCDA1^ utility by avoiding the *ung* deletion. To enhance UGI activity, we initially tested a new constitutive promoter for *ugi* or a triply fused protein, UGI_PmCDA1_T7RNAP. However, both were less efficient than the *Δung* strain (Supplementary Figure S3). Next, we optimized the ribosomal binding site (RBS) of *ugi* (26) (Figure 2C), and obtained a suppression frequency indistinguishable from that of the *Δung* strain (Figure 2D). Thus, we were able to avoid the *ung* deletion for efficient eMutaT7^PmCDA1^-mediated mutagenesis.

### Dual expression system introduces all transition mutations at comparable frequencies

We examined whether the two deaminases could simultaneously install both C:G→T:A and A:T→G:C mutations at similar frequencies. Initially, we tested two triple-fused proteins, PmCDA1_TadA-8e_T7RNAP and TadA-8e_PmCDA1_T7RNAP, in which two deaminases were attached to the N-terminus of T7RNAP in different orders (Figure 3A, **2** and **3**). Sequencing of clones after 20 rounds of *in vivo* hypermutation revealed that PmCDA1_TadA-8e_T7RNAP installed more A:T→G:C mutations (84%) than C:G→T:A (16%), whereas TadA-8e_PmCDA1_T7RNAP generated more C:G→T:A (96%) than A:T→G:C (4%) (Figure 3B and Supplementary Figure S4A). This result indicates that the deaminase closer to T7RNAP is more active. Shorter or longer linker lengths between enzymes did not significantly reduce the gap (Supplementary Figure S5).

Next, we tested the expression of two mutators, eMutaT7^PmCDA1^ and eMutaT7^TadA-8e^, from a single plasmid (Figure 3C, **4**–**7**). The pDae079 plasmid, in which the eMutaT7^TadA-8e^ gene is located in front of the eMutaT7^PmCDA1^ gene, yielded the same amounts of A:T→G:C (50%) and C:G→T:A (50%) mutations (*P* = 0.87; Figure 3D and Supplementary Figure S4B). In contrast, the pDae080 plasmid, which reversed the order of the two mutators, disproportionately generated C:G→T:A (85%) over A:T→G:C (15%) (*P* = 0.012; Figure 3D and Supplementary Figure S4B). As expected, weaker UGI expression without the optimized RBS significantly reduced C:G→T:A mutations in the wild-type strain (*P* = 0.0046; Figure 3D and Supplementary Figure S4B) but produced comparable numbers of mutations in the *Δung* strain (A:T→G:C, 38%; C:G→T:A, 62%; *P* = 0.37; Figure 3D and Supplementary Figure S4B). We thus selected pDae079 for eMutaT7^transition^, which on average installed 5.7 transition mutations in the 1269-bp gene during 80-hour *in vivo* hypermutation.

### High-throughput sequencing demonstrates that eMutaT7^transition^ introduces all transition mutations at similar frequencies

To further dissect the eMutaT7^transition^-mediated *in vivo* hypermutation, we used next-generation sequencing (NGS) to analyze the sequences of ∼3.3 kb DNA fragments around the target region from mixed pools of cells taken at cycle 0 (*n* = 1) or cycle 20 (*n* = 3). We found that, among all substitution types, all four transition substitutions were significantly accumulated at cycle 20 (Supplementary Figure S6); the adjusted average substitution frequencies (frequency differences between cycle 0 and cycle 20) were 0.28% for A→G, 0.22% for T→C, 0.046% for G→A, and 0.41% for C→T, respectively. We further dissected the 3.3 kb DNA into three regions—upstream, target gene, and downstream. Among them, the target gene showed the highest level of adjusted transition substitution frequencies (0.52%, 0.54% and 0.54%, respectively) than the upstream (0.023%, 0.023% and 0.021%) and the downstream (0.089% 0.090% and 0.088%) regions (Figure 4A and B). This result supports the gene-specific mutagenesis of eMutaT7^transition^. As previously observed with eMutaT7^PmCDA1^ (20), the downstream region showed higher leakages of gene targeting than the upstream region. Given the very low rifampicin resistance frequencies of eMutaT7^PmCDA1^ (20) and eMutaT7^TadA-8e^ (Supplementary Figure S1C), however, we believe that eMutaT7^transition^ does not generate high level of off-target mutations in the genome.

The average number of eMutaT7^transition^-mediated substitution in the target gene was 6.7 (1269 bp × 0.53%) in NGS analysis, closely recapitulating the result from Sanger sequencing (5.7 substitutions; Figure 3D, **5**). The high-throughput sequencing data also confirmed that eMutaT7^transition^ generates comparable amounts of A:T→G:C and C:G→T:A substitutions, whose average ratio was 1:0.93 (Figure 4B). Taken together, NGS analysis corroborated that eMutaT7^transition^ rapidly introduces all transition mutations on the target gene at comparable frequencies.

### Additional analyses demonstrate high mutational activity and target tolerance of eMutaT7^transition^

To further estimate the mutational activity of eMutaT7^transition^, we performed fluctuation analysis for the loss-of-function of *pheS*_A294G (29). We used two target plasmids in which the target gene is controlled by either T7 promoter or an unrelated constitutive promoter. The conversion of the *pheS*_A294G loss-of-function colony counts to loss-of-function mutation rate with the FALCOR webtool (31) resulted in 3.6 × 10^−5^ loss-of-function mutations per generation with T7 promoter and 2.4 × 10^−9^ loss-of-function mutations per generation without T7 promoter, indicating 15 000-fold increase of the mutation rate with the proper targeting of eMutaT7^transition^ (Figure 5A and Supplementary Figure S7). This result suggests that eMutaT7^transition^ indeed has a high mutational activity.

We also tested target tolerance of eMutaT7^transition^ by using different target genes in various genetic contexts. We initially performed the 20-cycle *in vivo* hypermutation of two additional genes (*malE* and *gfp*) encoding maltose binding protein (MBP) and green fluorescent protein (GFP), respectively, as well as *pheS*_A294G with or without eMutaT7^transition^. We found that these three genes contained comparable numbers of transition mutations (average 6.3, 7.2 and 5.0 substitutions in *pheS*_A294G, *malE* and *gfp*, respectively), whereas the *pheS*_A294G gene without eMutaT7^transition^ displayed no mutation (Figure 5B and Supplementary Figure S8A). This result suggests that the presence of *pheS*_A294G itself does not induce hypermutation and that the high mutational activity of eMutaT7^transition^ is not limited to our model gene, *pheS*_A294G. We also tested the conditions in which T7-controlled *gfp* is inserted in the chromosome (Figure 5C), *malE* and *gfp* are located in a single target plasmid (Figure 5D), or *malE* and *gfp* are positioned in a target plasmid and chromosome, respectively (Figure 5E). We found that three conditions lead to total 7.0, 4.5 and 6.0 mutations, respectively, in which the latter two showed mutations on both genes (Figure 5C–E and Supplementary Figure S8B–D). These results suggest that eMutaT7^transition^ can target multiple genes in different locations.

Although majority of these results showed comparable numbers of A:T→G:C and C:G→T:A substitutions (Supplementary Figure S8A–C), one experiment almost exclusively showed A:T→G:C (Supplementary Figure S8D), indicating that only eMutaT7^TadA-8e^ was active during hypermutation. Because we used the same DNA sequence of T7RNAP for two mutator genes, the deletional recombination of these two mutator genes might generate the eMutaT7^TadA-8e^-only mutator plasmid. Indeed, we found that the mutator plasmid obtained from the cycle #20 of this sample was shorter than the original eMutaT7^transition^ plasmid (Supplementary Figure S8E), suggesting that eMutaT7^transition^ needs to be improved for longer *in vivo* hypermutation experiments.

### eMutaT7^transition^ evolves TEM-1 with various transition mutations

**Figure 6.**
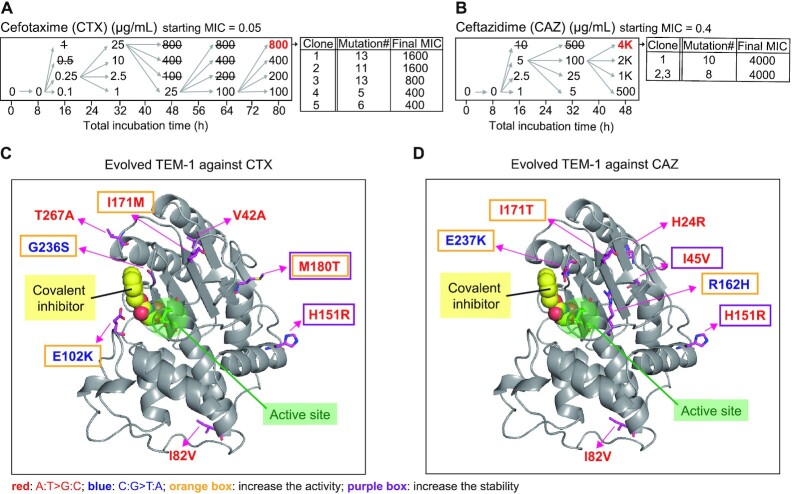
Continuous directed evolution of TEM-1 for antibiotic resistance using eMutaT7^transition^. (**A**, **B**) Evolutionary pathways of TEM-1 for resistance against CTX (A) or CAZ (B). Each number indicates an antibiotic concentration in a culture. Strikethrough indicates no growth. (**C, D**) Structure of TEM-1 (PDB, 1axb) showing a covalent inhibitor (yellow), the active site (green), A:T→G:C substitutions in evolved TEM-1 (red), and C:G→T:A substitutions in evolved TEM-1 (blue). Mutations in orange and purple boxes indicate those that increased enzyme activity and stability, respectively.

We previously demonstrated that eMutaT7^PmCDA1^ promoted rapid continuous directed evolution of TEM-1 for resistance against third-generation cephalosporin antibiotics, cefotaxime (CTX) and ceftazidime (CAZ) (20). Here, we tested eMutaT7^transition^ in the same way. We used the dual promoter/terminator approach to install both C→T and G→A mutations (17,20). By sequentially increasing antibiotic concentrations during multiple rounds of *in vivo* hypermutation, we elevated minimum inhibitory concentrations (MICs) from 0.05 to 400–1600 μg/ml in 80 h for CTX (Figure 6A) and from 0.4 to 4000 μg/ml in 48 h for CAZ (Figure 6B).

Sanger sequencing of several resistant clones revealed that their mutational spectra were more diverse than those obtained with eMutaT7^PmCDA1^ (Supplementary Figure S9). Adenine deamination (A:T→G:C) generated I45V, H151R, I171M, I171T and M180T, whereas cytosine deamination (C:G→T:A) generated E102K, R162H, G236S and E237K (Figure 6C, D, and Supplementary Figure S9). I45V, H151R and M180T have been reported to increase enzyme stability (43–46). Additionally, E102K, R162H, I171M/T, G236S and E237K are involved in resistance to CTX or CAZ (46–51). Other mutations are not found in clinical or laboratorial isolates (H24R and V42A), are found in the wild-type allele (I82V), or have unknown functional effect (T267A) (51). These results suggest that eMutaT7^transition^ indeed covers a wider protein mutational space for evolution.

In conclusion, this study described a new mutator system that combines eMutaT7^PmCDA1^ and eMutaT7^TadA-8e^, called eMutaT7^transition^. This new system has advantages over previous deaminase-T7RNAP mutators. First, eMutaT7^transition^ expands the mutational spectrum to all transition substitutions (C:G→T:A and A:T→G:C). eMutaT7^PmCDA1^ can mediate 8.4% of all amino acid changes (32 out of total 380 changes), but eMutaT7^transition^ expands them to 19% (74 changes). Accordingly, we observed in TEM-1 evolution experiments several A:T→G:C substitutions that have been previously identified in clinical or laboratorial isolates. Although transition substitutions nominally compose only a small fraction of all amino acid changes, they generally appear more frequently in natural variants, explaining approximately two-third of single nucleotide polymorphisms in several species (52–54). Second, all transition substitutions are produced at similar frequencies. This outcome was made possible by the use of two efficient deaminases, PmCDA1 and TadA-8e, along with appropriate expression of the two mutators and a DNA glycosylase inhibitor. In contrast, TRIDENT generated considerably more C:G→T:A substitutions (∼95%) in yeast (21).

Future research should aim to include transversion mutations in the mutational spectrum without significantly sacrificing substitution frequencies. Additionally, eMutaT7^transition^ would be improved to suppress the deletional recombination for longer *in vivo* hypermutation experiments; either the different DNA sequences of T7RNAP for two mutators or the recently reported TadA variants that can mutate both cytidine and adenine simultaneously (55,56) may enhance its property. With its good substitution frequencies and wider mutational spectrum, we believe that eMutaT7^transition^ or its improved variants can become the method of choice in synthetic biology studies requiring evolutionary approach, particularly in evolution or engineering of enzymes, metabolic pathways, or gene circuits.

## DATA AVAILABILITY

Illumina sequencing data have been deposited in the ArrayExpress database at EMBL-EBI (www.ebi.ac.uk/arrayexpress) under accession number E-MTAB-12258. Other data that support the findings of this work can be found in the paper and in the Supplementary Data files. Protein and primer sequences are listed in supplementary tables. All *E. coli* strains and plasmids described in this work are available upon request. The pDae029 (eMutaT7^TadA-8e^), pDae069 (eMutaT7^PmCDA1^), and pDae079 (eMutaT7^transition^) have been deposited and are available through Addgene (#187620 for pDae029; #187621 for pDae069; #187622 for pDae079).

## Supplementary Material

gkad266_Supplemental_Files

## Notes

Present address: Ga-eul Eom, CJ CheilJedang, CJ BLOSSOM PARK, 55, Gwanggyo-ro 42-beon-gil, Yeongtong-gu, Suwon-si, Gyeonggi-do 16495, Republic of Korea.
