# Supplementary material for: A dual gene-specific mutator system installs all transition mutations at similar frequencies in vivo
Source: Nucleic Acids Res. 2023 Apr 18;51(10):e59. doi: 10.1093/nar/gkad266 (PMC10250238; doi:10.1093/nar/gkad266)
Supplement: gkad266_Supplemental_Files [file gkad266_supplemental_files.zip › Supplementary Data 1.pdf]

A dual gene-specific mutator system installs all transition mutations at similar frequencies *in vivo*

Daeje Seo, Bonghyun Koh, Ga-eul Eom, Hye won Kim, and Seokhee Kim*

Seoul National University

Contents	Page
**Table of Contents**	S1
**Supplementary Figures**	S2-S10
**Supplementary Tables**	S11-14
**References**	S15

*Corresponding author: seokheekim@snu.ac.kr

A

clone#	induction	A>G	sub#	T>C	sub#	total#	average
1-1, 2, 3	+		0		0	0	0.0
2-1		-92*	1	-113*, -93*, -85*, -59*, -17*, R281R	6	7	
2-2	+	-98*, -92*, N26D	3	-17*, R186R	2	5	6.7
2-3		-98*, -92*, -60*, S16G, T91A	5	-59*, G33G, R186R	3	8	
3-1, 2, 3	+		0		0	0	0.0
4-1, 2, 3	+		0		0	0	0.0
5-1, 2, 3	-		0		0	0	0.0

Amino acids were numbered according to the protein sequence of *pheS*_A294G
A>G substitution on the codoing strand, **red**; **T>C** substitution on the coding strand, **orange**
* substitutions not made on *pheS*_A294G gene orf but made between a T7 promoter and a T7 terminator

B

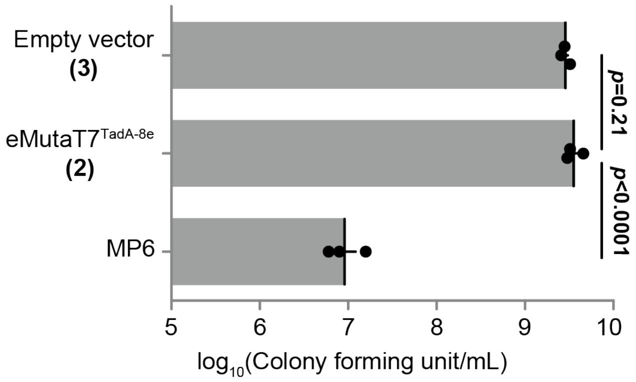

C

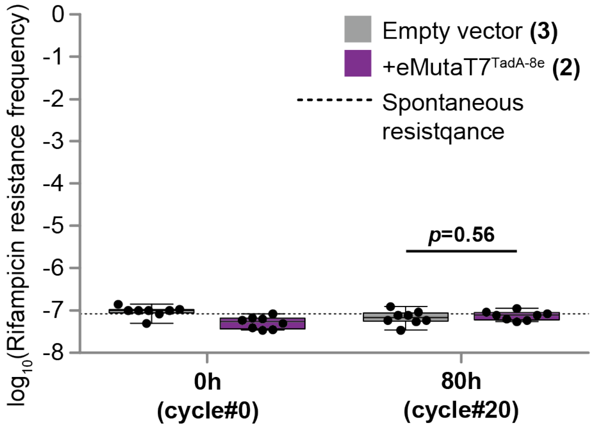

Supplementary Figure S1. Characterization of eMutaT7$^{TadA-8e}$. (A) A list of substitutions found in samples shown in Figure 1C. **(B)** Viability of cells expressing no protein (setup **3** in Figure 1A), eMutaT7$^{TadA-8e}$ (setup **2** in Figure 1A), or MP6. Data are presented as dot plots with mean ± standard deviation (SD) (n = 3). *P* values were obtained using two-tailed Student's t-test; p < 0.05 was considered significant. **(C)** Off-target mutation level of cells expressing eMutaT7$^{TadA-8e}$ (setup **2** in Figure 1A) or no protein (setup **3** in Figure 1A) was estimated by rifampicin resistance frequency. The dotted line represents spontaneous rifampicin resistance level. Box limits indicate interquartile range; whiskers, minimum to maximum; center line, median; dots, individual data points (n = 8). *P* values were obtained using a two-sided Mann-Whitney test; p < 0.05 was considered significant.

A

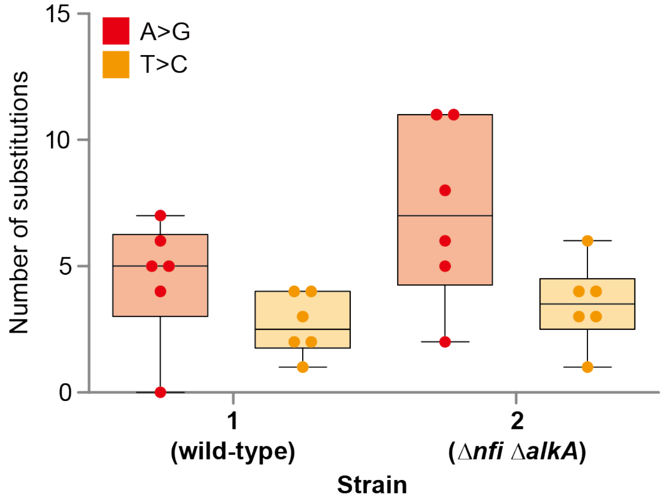

B

clone#	A>G	sub#	T>C	sub#	total#	average
1-1		0	-144*, D20D, Y31H, R111R	4	4	7.2
1-2	-112*, -95*, -92*, -63*,	4	-59*, -17*	2	6	
1-3	-65*, -41*, S2S, N139D, Q200R	5	-64*, -17*, G33G, R167R	4	9	
1-4	-98*, -32*, Y31C, Q69R, T251A	5	-64*	1	6	
1-5	-92*, -81*, -63*, T126A, Y198C, T216A, Q240R	7	G33G, H138H	2	9	
1-6	-92*, -63*, Y31C, K34E, S314S, +11*	6	-17*, V109V, R167R	3	9	
2-1	-133*, -131*, -65*, -63*, N196D	5	D25D, Y137H, F326L	3	8	11
2-2	-131*, -109*, -98*, -92*, -6*, V173V, T216A, X328X	8	-87*, -17*, L24S, A81A	4	12	
2-3	-133*, -131*, -98*, M1V, N26D, E102E	6	-17*	1	7	
2-4	-32*, T221A	2	-134*, -93*, -83*	3	5	
2-5	-131*, -98*, -82*, -65*, -63*, M1V, N26D, H138R, T216A, S314S, +76*	11	-17*, Y31H, F125F, Y194H	4	15	
2-6	-131*, -98*, -65*, M1V, Y31C, K34E, E51E, I145V, T226A, Y247C, +99*	11	-66*, -17*, -1*, I15I, N72N, R74R	6	17	

Amino acids were numbered according to the protein sequence of *pheS*_A294G
A>G substitution on the codoing strand, **red**; **T>C** substitutionon the coding strand, **orange**
* substitutions not made on *pheS*_A294G gene orf but made between a T7 promoter and a T7 terminator

Supplementary Figure S2. The deletion of inosine glycosylases did not significantly elevate the mutation frequency of eMutaT7$^{TadA\text{-}8e}$. (A) Number of A>G (red) and T>C (orange) substitutions in the wild-type (setup **1** in Figure 2B) or Δ*nfi* Δ*alkA* (setup **2** in Figure 2B) strains. Box limits indicate interquartile range; whiskers, minimum to maximum; center line, median; dots, individual data points (n = 6). **(B)** A list of substitutions found in (A).

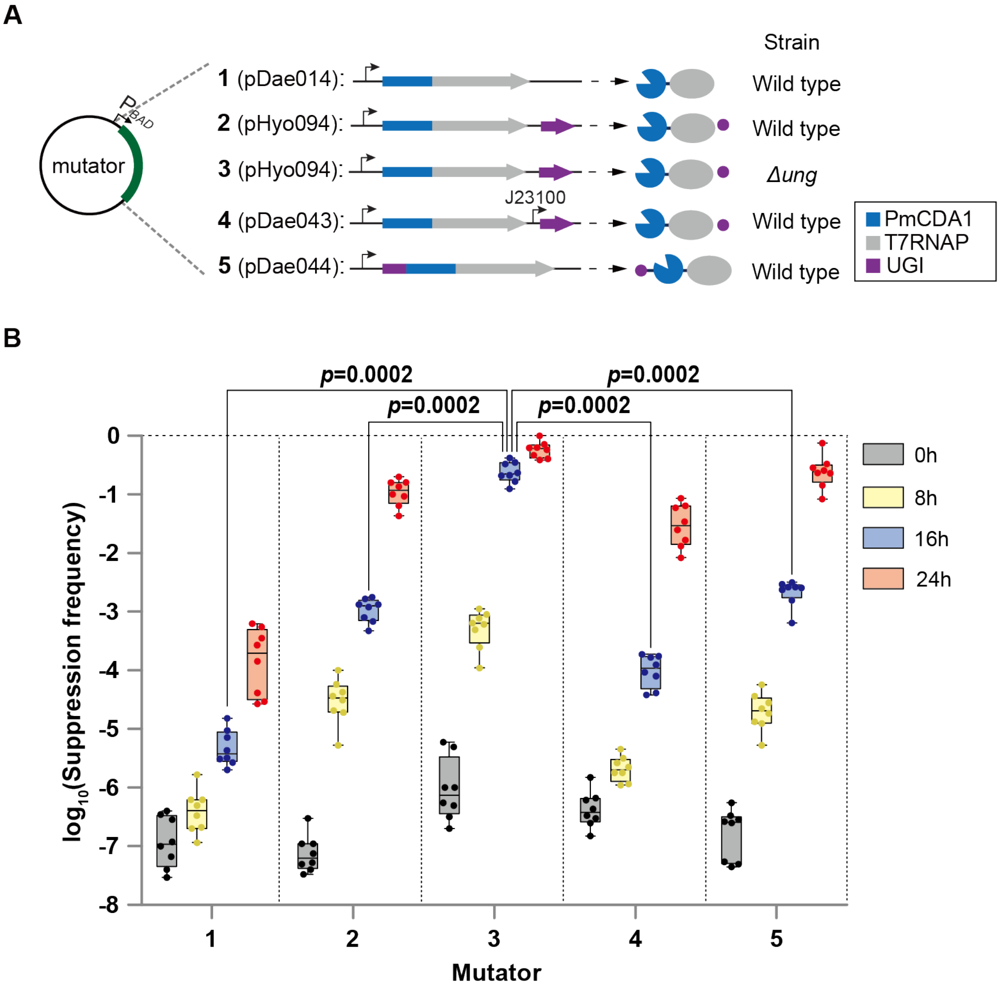

Supplementary Figure S3. eMutaT7^{PmCDA1} optimization. (A) Five tested conditions for eMutaT7^{PmCDA1}-mediated hypermutation: no UGI in wild-type strain (**1**), UGI expression under the original promoter in wild-type (**2**) or Δ*ung* (**3**) strains, UGI expression under a new constitutive promoter (J23100) in wild-type strain (**4**), and a triple fusion of UGI, PmCDA1, and T7RNAP in the wild-type strain (**5**). **(B)** Frequency of the *pheS*_A294G toxicity suppression at each mutagenesis cycle for cells evolved under the five different conditions (**1-5**) shown in (A). Box limits indicate interquartile range; whiskers, minimum to maximum; center line, median; dots, individual data points (n = 8). *P* values were obtained with two-sided Mann-Whitney tests; $p < 0.05$ was considered significant.

A

clone#	A:T>G:C	sub#	C:G>T:A	sub#	total#	average
1-1, 1-2, 1-3, 1-4, 1-5, 1-6		0		0	0	0
**clone#**	**A:T>G:C**	**sub#**	**C:G>T:A**	**sub#**	**total#**	
2-1	-113*, -92*, -87*, -83*, -77*, -65*, -63*, -32*, -17*, M1A(ATG to GCG), S19S, D20D, Y31C, K34E, H37R, I60V, I145V, R167R	19	Q181X	1	20	
2-2	-65*, -64*, -32*, -17*, V21V, D25D, T112A, T221A, Y247C	9	-58*, -45*, A9A, A18V, R74C, F295F	6	15	
2-3	-98*, -65*, -64*, -59*, -17*, G33G, T221A, G264G, V269A	9		0	9	
2-4	-134*, -92*, -85*, -77*, -63*, -59*, D25D, N26D, Y31H, E132E, I145V, I241V, V279A	13	+32*, +49*	2	15	14
2-5	-96*, -59*, -17*, V8A, K34E, X328X	6		0	6	
2-6	-94*, -92*, -69*, -63*, -30*, M1V, S10S, Y31C, V59V, I145V, D161G, K179E, Y247C, K324K	14	-71*, I60I, G105S, T216T	4	18	
**clone#**	**A:T>G:C**	**sub#**	**C:G>T:A**	**sub#**	**total#**	
3-1		0	-39*, Q41X, T129T, Q169X, Q182X, Y198Y, +13*	7	7	
3-2	-17*	1	-33*, L40F, Q41X, T44T, L78L, Q208X	6	7	
3-3		0	-58*, L7L, A13A, V29V, L40F, R111C, T126T, P150L, R176C, T221T, R242C	11	11	8.2
3-4		0	-58*, G36R, L40F, I60I, Q69X, R176C, Q208X, S254F, F316F, +32*	10	10	
3-5		0	-10*, -7*, -3*, Q66X, I113I, Q169X, +31*	7	7	
3-6	Y137H	1	N61N, Q69X, I113I, T129T, A255V, L323L	6	7	

B

clone#	A:T>G:C	sub#	C:G>T:A	sub#	total#	average
4-1		0	Q41X, L78L	2	2	
4-2	-91*	1	A13V, Q41X, T221T, Q240X, +3*, +32*	6	7	
4-3	E300G	1	Q17X, F295F	2	3	
4-4	-143*, -96*, T216A	3	-117*, -42*, A63V, Q69X, R111C, Q169X, R176C, S254F	8	11	5.5
4-5		0	-42*, Q17X, H138Y, L320L	4	4	
4-6		0	-42*, -33*, S2L, Q41X, T126T, R281C	6	6	
5-1	-98*, -17*	2	-71*, P277S	2	4	
5-2	-17*, N26D	2	Q41X, T126I, T251I, P277S	4	6	
5-3	-92*, -17*, N26D, Y31H, V279A	5		0	5	
5-4	-17*, Y247C, V279A	3	L4L, Q69X, A180A	3	6	5.7
5-5	D20D, Y137H	2	-97*, -68*, Q41X, H138Y, Q169X, T310T, +48*	7	9	
5-6	F125F, I145V, N281D	3	Q41X	1	4	
6-1	-17*, N26D, L32L, I145V	4		0	4	
6-2	-66*, -59*	2		0	2	
6-3	-98*, -92*, -87*, -73*, -64*, -2*, N26D, Y31C	8		0	8	
6-4	S2S	1	Q41X	1	2	4.5
6-5	-144*, Y31C, R115R, N139D	4		0	4	
6-6	-109*, -92*, -76*, -17*, N26D, N72N, Y198C	7		0	7	
7-1	V109V	1	-58*, -7*, -4*, T251T	4	5	
7-2	-17*, D25G, T112A	3	A22A, V29V, Q41X, Q169X, T177T, Q181X, R186C, T251T, F293F	9	12	
7-3	M1T	1	-58*, -22*, R115C, I133I, H138Y, R195C	6	7	7.5
7-4	-92*, -32*, -17*, N26D, +20*	5		0	5	
7-5	-144*, -17*, S2S, N61D, R99R, R232R	6	L40F, Q41X, R164C, T241T	4	10	
7-6	-98*	1	D25N, Q41X, P108L, R244C, +22*	5	6	

Amino acids were numbered according to the protein sequence of *pheS*_A294G

A>G substitution on the codoing strand, **red**; **T>C** substitution on the coding strand, **orange**; **C>T** substitution on the coding strand , **blue**; **G>A** substitution on the coding strand, **cyan**

* substitutions not made on *pheS*_A294G gene orf but made between a T7 promoter and a T7 terminator

Supplementary Figure S4. A full list of substitutions in samples shown in Figure 3B (A) and 3D (B).

The first numeric values indicate the setup numbers shown in Figure 3A (**1-3**) or 3C (**4-7**).

A

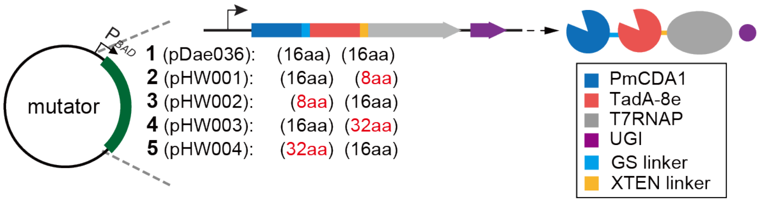

B

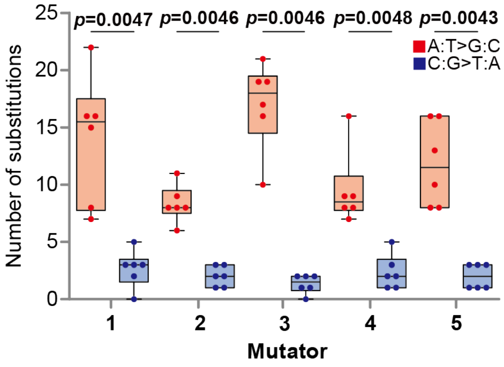

C

clone#	A:T>G:C	sub#	C:G>T:A	sub#	total#
1-1	-131*, -111*, -109*, -93*, -92*, -64*, -17*, D25D, Y31H, H148R, T162T, T170A, E210G, T251A, S314S	15	-33*, Q41X, T216T	3	18
1-2	-98*, -92*, -65*, -64*, -30*, -17*, -1*, E6E, S10G, V21V, D25G(GAT to GGC), Y31R(TAT to CGT), Y198C, K265K	16	-71*, F140F, F158F	3	19
1-3	-92*, -59*, -17*, S2P, N26D, T39A, M42T, A56A, T112A, D114G, I145V, F160L, V173V, R186C, N233S, K324K	16	A5V, I60I, P150S, R186C, P277L	5	21
1-4	-131*, -106*, -98*, -59*, -32*, -17*, I188V	7		0	7
1-5	-131*, -92*, -90*, -17*, Y31H, K64K, Y194H, K262R	8	P49L, Q69X, L96L	3	11
1-6	-113*, -109*, -96*, -95*, -93*, -87*, -17*, N26D, Y31H, K35R(AAA to AGG), R74R, E102G, H138R, I145V, H148R, T162T, R186R, T203A, L223P, F250F, G264G	22	A153T, +19*	2	24
2-1	-92*, -63*, -17*, D25D, V109V, V173V, E236G, +56*	8	-70*, -36*, V29V	3	11
2-2	-108*, -92*, -59*, G121G, I145T, F220F, +20*, +117*	8	A9A, T251T	2	10
2-3	-59*, -17*, N61D, L83P, Y137C, +23*	6	T251T	1	7
2-4	-109*, -92*, -54*, -17*, Y31C, E51G, R74R, H148R, T157A, T221A, Y290C	11	-58*, T221A, +32*	3	7
2-5	-92*, -82*, -77*, -67*, -54*, -17*, Y31C, T216A	8	-58*, S2L	2	7
2-6	-63*, -59*, -17*, D20D, D25D, T42A, R111R, Y137H, T251A	9	-97*	1	7
3-1	-133*, -93*, -92*, -85*, -66*, -59*, -44*, -17*, N26D, H37R, I145V, T157A, V173A, Q182R, N233S, Y290H	16	L4L, T126T	2	18
3-2	-98*, -92*, -17*, H3H, I15I, N26D, N72N, F125F, T216A, V269A	10	-71*	1	11
3-3	-92*, -89*, -82*, -81*, -66*, -64*, -59*, -17*, V8V, S16G, G33G, R74R, N84N, I113V, R167R, V173A, I218V	17	G271S	1	18
3-4	-144, -92*, -65*, -63*, -17*, -1*, S16G, V21V, N26D, G33G, H37R, R74R, T126A, T129A, I145V, F250F, K262E, G264G, S314S, X328X, +97*	21	V29V, T251I	2	23
3-5	-83*, -82*, -75*, -65*, -64*, -17*, M1T, S10S, N26D, V27A, Y31C, V67A, N72N, V109V, F125F, H138H, R167R, Y247C, G264G	19		0	19
3-6	-144*, -112*, -98*, -92*, -64*, -59*, -17*, L24L, N26D, Y31C, G33G, I145V, G147G, V173A, Q208R, V214A, Y247C, T251A, G264G	19	Q169X, T216T	2	21
4-1	-131*, -54*, -17*, D20D, R11R, E117E, F125F, T126A, Y137H	9	P49L	1	10
4-2	-109*, -92*, -77*, -65*, -17*, -6*, -2*, -1*, M1A(ATG to GCG), V21V, D25D, H37R, T91A, Y137C, T168A	16	-110*, N61N, R176C, Q181X, P277L	5	21
4-3	-131*, -17*, K34E, Y198C, Y247C, T251A, K262E, S314S, +23*	9	-71*, L231L	2	11
4-4	-17*, N26D, Y31C, G33G, I145V, Y194C, Y198C	7	Q66X, R115C	2	9
4-5	-77*, -66*, -17*, V21V, D25D, I145V, D154G, M304T	8	-58*, S2L, Q66X	3	11
4-6	-131*, -82*, -64*, -17*, S2S, D25D, +23*, +43*	8	Q41X	1	9
5-1	-98*, -95*, -83*, -82*, -66*, -59*, -17*, L24L, N26N, Y31C, A82A, H138R, I145A(ATT to GCT), T168A, H228R	16	Q69X	1	17
5-2	-131*, -98*, -96*, -77*, -51*, -17*, G33G, L38L, T112A, T126A, T163A, T177A, I285V	13	-68*, Q41X, A58A	3	16
5-3	-131*, -98*, -93*, -65*, -60*, -17*, -2*, I145V, H148R, V279A	10	-48*, M42I, Q182X	3	13
5-4	-131*, -92*, -63*, -47*, -30*, -17*, T221A, V269A	8	S291F	1	9
5-5	-98*, -94*, -93*, -91*, -66*, -64*, -59*, -17*, V21V, D25D, K34E, H155R, D215D, D286G, X328Q, +40*	16	I113I	1	17
5-6	-133*, -113*, -93*, -78*, -30*, -17*, N26D, V27A	8	Q69X, T221T, G292S	3	11

Amino acids were numbered according to the protein sequence of *pheS*_A294G

A>G substitution on the codoing strand, **red**; **T>C** substitution on the coding strand, **orange**; **C>T** substitution on the coding strand , **blue**; **G>A** substitution on the coding strand, **cyan**

* substitutions not made on *pheS*_A294G gene orf but made between a T7 promoter and a T7 terminator

Supplementary Figure S5. Linker optimization of a triple fusion protein, PmCDA1_TadA-8e_T7RNAP. (A) Design of variants with different linkers: two original linkers (**1**), a shorter linker between TadA-8e and T7RNAP (**2**), a shorter linker between PmCDA1 and TadA-8e (**3**), a longer linker between TadA-8e and T7RNAP (**4**), a longer linker between PmCDA1 and TadA-8e (**5**). **(B)** Number of substitutions found in six clones from samples at 20 mutagenesis cycle. Numeric labels in the x-axis (**1-5**) indicate five different mutators shown in (A). Box limits indicate interquartile range; whiskers, minimum to maximum; center line, median; dots, individual data points (n = 6). *P* values were obtained with two-sided Mann-Whitney tests; $p < 0.05$ was considered significant. **(C)** A list of substitutions found in samples shown in (B).

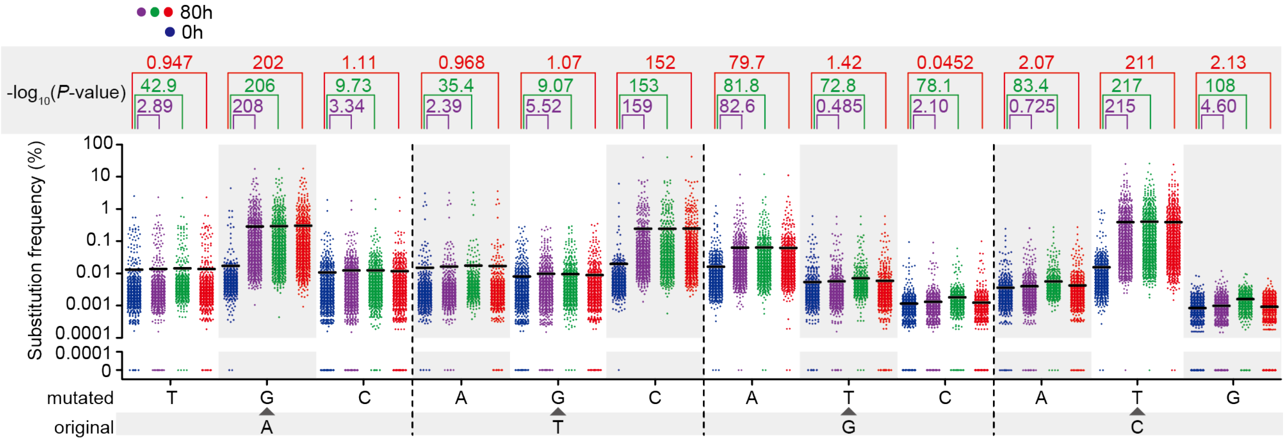

Supplementary Figure S6. Illumina sequencing demonstrates that eMutaT7^{transition} introduces all transition substitutions. Substitution frequency of all possible substitution types by eMutaT7^{transition} at 0 h (n = 1; blue) and 80 h (n = 3; purple, green, and red). Y-axes above and below 0.0001% are in log- and linear-scale, respectively. Data are presented as dot plots with averages (short black lines). *P* values were obtained with two-sided Mann-Whitney tests and presented as -log$_{10}$(*P* value).

A

Experiment number	eMutaT7[transition]	T7 Promoter	The number of total cell (N_t)	The number of mutations (m)	Mutation rate (μ)	95% UB	95% LB
1	+	+	536250	23.07	4.30×10^{-5}	5.11×10^{-5}	3.55×10^{-5}
2	+	+	1112500	36.06	3.24×10^{-5}	3.77×10^{-5}	2.74×10^{-5}
3	+	+	625000	20.72	3.32×10^{-5}	3.96×10^{-5}	2.71×10^{-5}
4	+	-	6675000000	15.56	2.33×10^{-9}	2.83×10^{-9}	1.87×10^{-9}
5	+	-	3832500000	6.076	1.59×10^{-9}	2.04×10^{-9}	1.17×10^{-9}
6	+	-	9930000000	33.55	3.38×10^{-9}	3.94×10^{-9}	2.85×10^{-9}

B

Experiment number		The number of mutants (r)																							
	Culture number	1	2	3	4	5	6	7	8	9	10	11	12	13	14	15	16	17	18	19	20	21	22	23	24
1		76	127	164	119	128	91	198	163	8	108	75	29	100	72	58	139	102	73	67	160	97	80	180	162
2		84	276	43	164	204	408	172	180	61	176	148	280	284	144	7	21	512	256	47	144	188	172	224	176
3		87	102	143	74	203	37	126	39	30	51	5	138	90	44	113	102	141	180	7	348	70	6	101	43
4		216	516	50	612	576	408	540	492	12	180	9	516	528	30	65	348	51	59	12	12	34	3	2	7
5		6	8	70	8	11	55	4	22	9	1	0	60	125	19	20	52	39	58	18	20	26	16	5	5
6		12	15	58	87	408	420	46	95	1524	1884	516	324	264	228	20	25	10	444	552	360	348	25	14	34

Supplementary Figure S7. Estimation of *pheS*_A294G loss-of-function mutation rates by fluctuation analysis. (A) The experimental data of fluctuation analysis that represents the total cell counts, the number of loss-of-function mutations per culture, the loss-of-function mutation rates obtained by FALCOR webtool, and their 95% confidence intervals. **(B)** The experimental data of fluctuation analysis that represents the observed number of loss-of-function mutants in a culture**.**

A

clone#	A:T>G:C	sub#	C:G>T:A	sub#	total#	average
1-1		0		0	0	0
1-2		0		0	0	
1-3		0		0	0	
1-4		0		0	0	
1-5		0		0	0	
1-6		0		0	0	
2-1	-89*, D25D	2	-58*, G124S	2	4	6.3
2-2	-93*, I15I, G33G, Y194C	4	Q41X	1	5	
2-3	-65*	1	-61*, L7L, Q41X, A88V, L96L, T112T, I113I, T126T, P184L, T251T	10	11	
2-4	-131*, -66*, D25D	3	Q208X	1	4	
2-5		0	V29V, Q41X, I60I, W159X, T163T, R176C	6	6	
2-6	-64*, -17*, D25D, N139D, I145V, +90	6	-61, Q41X	2	8	
3-1	-109*, -59*, -27*, Y172H, E173G, I334V	6	P126L, P134L	2	8	7.2
3-2	-17*, I34I	2		0	2	
3-3	-98*, -92*, -17*, Y172H	4	-115*, W11X, P41L, A97A, T129T, P134S, T209T, F251F, +32	9	13	
3-4	-92*, -89*, +97*	3	-58*, -55*, T32T, T37T, P108L, P155L	6	9	
3-5	T3T	1	-58*, N13N, D42N, P134S, D198N, S234S	6	7	
3-6		0	-130*, -128*, -70*, -25*	4	4	
4-1	-92*, -17*, V94V, I137V, H149R, N160D	6		0	6	5.0
4-2	-32*, N24D, V94A	3	-25*, N106N, +10	3	6	
4-3	-77*, -64*, -17*	3	-4*, -3*, F65F, P76L, F101F, I124I, Q184X, S209L, +75	9	12	
4-4	-17*, +20*	2	+31*	1	3	
4-5	-17*	1	F166F	1	2	
4-6	I15V	1		0	1	

B

	GFP					
clone#	A:T>G:C	sub#	C:G>T:A	sub#	total#	average
1	-95*, -64*, G41G, G52G, C71R, Y152H	6	P212P	1	7	7.0
2	I137V, Y183C	2	-68*, V113V, I124I, M154I, P197L, L202L, P212L, +32*	8	10	
3	E112E, R123G, S148S	3	Q70X, S73L, P90P, T98I, P188S	5	8	
4	-92*, T10A, Y40C, K46K, R123G	5		0	5	
5	T51A, Y238C	2	P76L, F85F, A180V, P197L, +10*	5	7	
6	-17*	1	-3*, D104D, P197L, +3*	4	5	

C

	MBP				GFP					
clone#	A:T>G:C	sub#	C:G>T:A	sub#	A:T>G:C	sub#	C:G>T:A	sub#	total#	average
1	-32*, -17*, T129A	3		0	K4E, L54L	2		0	5	4.5
2	Y172H, Y342H	2	T129T, F170F, R345C	3		0	I124I	1	6	
3	K35E, K252E, Q366R	3		0	T63A	1		0	4	
4	-92*, T250A	2	N350N	1		0	I124I	1	4	
5	K7E	1		0	R123G	1		0	2	
6	N19S	1	W11X, N228N	2	V151V	1	P14S, +32*	2	6	

D

	MBP				GFP					
clone#	A:T>G:C	sub#	C:G>T:A	sub#	A:T>G:C	sub#	C:G>T:A	sub#	total#	average
1	-17*, A64A	2		0	H78H	1		0	3	6.0
2	-30*, -17*, G144G, V245A, +88*	5		0	-126*, -32*, S87S, I129V, Y152H	5	I124I	1	11	
3	-114*, -98*, -17*, T54A, G266G, K296E	6		0	-92*, G21G	2		0	8	
4	-17*, M1V	2		0	G128G, Y146C	2		0	4	
5	A64A, +8*	2		0	-93*, -17*, P14P, G68G	4		0	6	
6	G69G, Y107C	2		0	-2*, T51A	2		0	4	

Amino acids were numbered according to the protein sequence of target genes (*pheS*_A294G, MBP or GFP)
A>G substitution on the codoing strand, **red**; **T>C** substitution on the coding strand, **orange**; **C>T** substitution on the coding strand , **blue**; **G>A** substitution on the coding strand, **cyan**
* substitutions not made on target genes (*pheS*_A294G, MBP or GFP) orf but made between a T7 promoter and a T7 terminator
[1]substitutions per 1kb per day

E

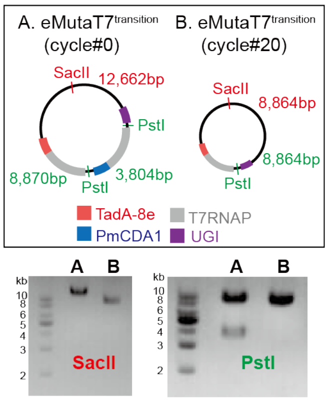

Supplementary Figure S8. A full list of substitutions in samples shown in Figure 5B (A), 5C (B), 5D (C), and 5E (D). The first numeric values indicate the setup numbers shown in Figure 5B (**1-4**). **(E)** DNA length of eMutaT7^{transition} plasmid at cycle#0 or cycle#20 restricted by SacII or PstI enzyme.

A Mutation list of evolved TEM-1 against cefotaxime (CTX)

Clone #	A>G	sub#	T>C	sub#	C>T	sub#	G>A	sub#	total#	MIC (µg/mL)
1	-32*, -22*, M1M***, I11V**, E62E, I82V, **H151R**, K213K	8	V42A, **M180T**	2		0	-81*, **E102K**, **G236S**	3	13	1600
2	-32*, E62E, I82V, H151R	4	F22S**, V42A, **M180T**, G242G	4		0	-81*, **E102K**, **G236S**	3	11	1600
3	-32*, E62E, I82V, **H151R**, **I171M**	5	F22S**, V42A, L160L, **M180T**, +63*	5		0	-81*, **E102K**, **G236S**	3	13	800
4	**H151R**	1	V42A, **M180T**	2		0	-81*, **G236S**	2	5	400
5	**H151R**, T267A	2	-25*, **M180T**	2		0	-81*, **G236S**	2	6	400
								average	9.6	

B Mutation list of evolved TEM-1 against ceftazidime (CAZ)

Clone #	A>G	sub#	T>C	sub#	C>T	sub#	G>A	sub#	total#	MIC (µg/mL)
1	-32*, A16A**, I82V, **H151R**	4	-33*, -25*, S128S, N272N	4		0	**R162H**, **E237K**	2	10	4000
2, 3	-32*, H24R, **I45V**, **H151R**	4	**I171T**, N272N	2		0	**R162H**, **E237K**	2	8	4000
								average	8.7	

Amino acids were numbered according to the protein sequence of TEM-1 including signal sequence.
Silent mutations, **grey**; mutations which increse the MIC of CTX and CAZ, **orange**;
mutations that increases th enzyme stability, **purple**
* substitutions not made on TEM-1 gene orf but made between a T7 promoter and a T7 terminator
** substitutions made on signal sequence (23AA)
*** substitution on start codon from ATG to GTG

Supplementary Figure S9. A full list of mutations found in samples shown in Figure 6A (A) and 6B (B).

Supplementary Table S1. *E. coli* strains used in this study

Strain	Description	Reference
DH5α	F^{-} *endA1 glnV44 thi-1 recA1 relA1 gyrA96 deoR nupG purB20* φ80d*lacZ*ΔM15 Δ(*lacZYA-argF*)U169, hsdR17($r_K^{-}m_K^{+}$), λ^{-}	(1)
W3110	F^{-} λ^{-} rph-1 INV(rrnD, rrnE)1	(2)
cHYO057	W3110 *Δung::kan*^{R}	(2)
cDJ085	W3110 *ΔalkA::Sm*^{R} *Δnfi::Kan*^{R}	This study
cDJ092	W3110 *ΔlacZ::Kan*^{R}-P_{T7}-*gfp*-T_{T7}	This study

Supplementary Table S2. Plasmids used in this study

Plasmid	Construct	Description	reference
pBAD33		Experimental control/cloning vector	
pHyo094	pBAD33-PmCDA1-T7RNAP, *ugi*	eMutaT7^{PmCDA1}, source of PmCDA1	(2) (addgene # 173124)
pHyo182	pVS133-lacI, *pheS*_A294G	Target plasmid (pheS_A294G)	(2) (addgene # 173136)
pHyo183	pBAD33-T7RNAP, *ugi*	control	(2)
pHyo245	pVS133-dualT7_*pheS*_A294G	Dual promoter system/cloning vector	(2) (addgene # 173147)
pREMCM3		Tetracycline resistance gene	(3)
TadA_mut	BamH1-GS-TadA*-GS-EcoR1	Source of TadA-7.10 gene	(4)
MP6		Mutator for PACE	(5)
pDae027	pCDF-TadA-8e-TadA-8e	Source of TadA-8e gene	(6)
pDae014	pBAD33-PmCDA1-T7RNAP	eMutaT7^{PmCDA1} without *ugi*	This study
pDae028	pBAD33-TadA-7.10-T7RNAP	eMutaT7$^{TadA\text{-}7.10}$	This study
pDae029	pBAD33-TadA-8e-T7RNAP	eMutaT7$^{TadA\text{-}8e}$	This study (addgene # 187620)
pDae032	pBAD33-TadA-8e	Control	This study
pDae035	pBAD33-TadA-8e-T7RNAP, PmCDA1-T7RNAP, ugi	the expression of two mutators, eMutaT7$^{TadA\text{-}8e}$ and eMutaT7^{PmCDA1}	This study
pDae036	pBAD33-PmCDA1-TadA-8e-T7RNAP, *ugi*	triply-fused protein of PmCDA1, TadA-8e, and T7RNAP	This study
pDae037	pBAD33-TadA-8e-PmCDA1-T7RNAP, *ugi*	triply-fused protein of TadA-8e, PmCDA1 and T7RNAP	This study
pDae043	pBAD33-PmCDA1-T7RNAP, J23100(promoter), *ugi*	Constitutive promoter for *ugi*	This study
pDae044	pBAD33-ugi-PmCDA1-T7RNAP	triply-fused protein of *ugi*, PmCDA1, and T7RNAP	This study
pDae069	pBAD33-PmCDA1-T7RNAP, (RBS) *ugi*	eMutaT7^{PmCDA1} with optimized RBS	This study (addgene # 187621)
pDae079	pBAD33-TadA-8e-T7RNAP, PmCDA1-T7RNAP, (RBS) *ugi*	eMutaT7^{transition}	This study (addgene # 187622)
pDae080	pBAD33-PmCDA1-T7RNAP, (RBS) *ugi*, TadA-8e-T7RNAP	the expression of two mutators, eMutaT7^{PmCDA1} and eMutaT7$^{TadA\text{-}8e}$ and optimized *ugi*	This study
pHW001	pBAD33-PmCDA1-16aa-TadA-8e-8aa-T7RNAP, *ugi*	Shoter XTEN linker	This study
pHW002	pBAD33-PmCDA1-8aa-TadA-8e-16aa-T7RNAP, *ugi*	Shoter GS linker	This study
pHW003	pBAD33-PmCDA1-16aa-TadA-8e-32aa-T7RNAP, *ugi*	Longer XTEN linker	This study
pHW004	pBAD33-PmCDA1-32aa-TadA-8e-16aa-T7RNAP, *ugi*	Longer XTEN linker	This study
pGE158	pVS133-dualT7_ss-TEM-1, tetR	Evolution target (TEM-1)	This study
pDae117	pVS133-lacI, *malE*	Target plasmid (*malE* (Maltose binding protein))	This study
pDae118	pVS133-lacI, *gfp*	Target plasmid (*gfp* (Green fluorescent protein))	This study
pDae119	pVS133-lacI, *malE*, *gfp*	Target plasmid (*malE* and *gfp*)	This study
pDae120	pVS133-lacI, BBa_J23100, *pheS*_A294G	Target plasmid (*pheS*_A294G) controlled by unrelated constitutive promoter	This study
pDae123	pVS133-lacI, Kan^R, P_{T7}-*gfp*-T_{T7}	*gfp* gene with Kanamycin resistance gene	This study

Supplementary Table S3. Primers used in this study

Oligonucleotides	Sequence (5'→3')	Description
T7promoter	TAATACGACTCACTATAGGG	Universal sequencing primer
T7terminator	GCTAGTTATTGCTCAGCGG	
pBAD-F	ATGCCATAGCATTTTTATCCA	
pBAD-R	GATTTAATCTGTATCAGG	
022_PxUgT_PxT_ovlp_fw	AACACGATTAACATCGCTAAGAACG	Cloning of pDae029
029_pBAD_Gibson_rv	CCATGGTGAATTCCTCCTGAGCTCG	Cloning of pDae028
052_T7RNAPQ265_rv	GTTGACGCTCAAACATCTTGC	
053_PmCDA_P57_rv	GGGCTTGTTGACGGCATAG	
054_PmCDA_G137_fw	GGACTCTGGAATCTGAGGG	
055_UPT-GS_ovlp_fw	GTAGCGGCTCTGGTTCCGGCTCTGGTAGCGGATCCACAGACGCCGAGTACGTG	Cloning of pDae037
058_PUT2_GS_ovlp_rv	CCGGAACCAGAGCCGCTACCAGAGCCGGAACCAACGGCTGGAGACTTAGTGG	Cloning of pDae036
062_pYH103_ovlp1_rv	ATGCCATGGTGAATTCCTC	Cloning of pDae037
063_PmCDA_ovlp1_fw	CTCAGGAGGAATTCACCATGGC	
128_pYH103_dpolI_rv	CATATGCCATGGTGAATTCCTC	Cloning of pDae029
129_nfi_fw	GGTCACGGCATTCATCAGG	
130_nfi_rv	GACATGCTGCCAGCTTTCC	
131_alkA_fw	GCGAAATGTTGCCGTCGC	
132_alkA_rv	CCCATCGCCTGATGCGAC	
133_TadA8e_IVA_fw	CTCAGGAGGAATTCACCATGGCATATGAGTGAAGTTGAATTCAGCCATG	Cloning of pDae029
134_TadA8e_IVA_rv	GAAGTCGTTCTTAGCGATGTTAATCGTGTTACTTTCGGGTGTGGCG	Cloning of pDae029
137_TadAdimer_del_fw	GGTTCCGGTAGCTTGTCTGAAGTC	Cloning of pDae028
138_TadA_del_fw	CATATGTCTGAAGTCGAATTTAGCCACG	Cloning of pDae028
161_dalkA_SmR3_fw	ATGGCGGCAAAATTGACCGCCAGAGTGGCACAGCTTTATGGCGACCGAGTGAGCTAGCTATTTG	Amplification of streptomycin resistance gene from pCDF for construction of alkA k/o strain
162_dalkA_SmR3_rv	GGGAAGCAGATATACTCCGGAAAATCATCCAGCCGTTCGCGAACGAATTGTTAGACATTATTTGCC	
163_Am7(8e)_dL_rv	GGAGTCTCGCTGCCGCTTAATTAATGCTG	Cloning of pDae032
165_Am7(8e)_dT7_fw	GGACTTCGCGTTCGCGTAA	
170_duet_Am7_IVA2_fw	CAGAATTTGCCTGGCGGCAGACTTTTCATACTCCCGCCATTCAGAGAAG	Cloning of pDae035, pDae079 (=eMutaT7^{transition}), and pDae080
171_duet_tm_Am7_IVA1_rv	CAGGGTTATTGTCTCATGAGCG	
172_duet_PxT_IVA2_rv	CTGCCGCCAGGCAAATTC	
173_duet_tm_PxT_IVA1_fw	GTATCCGCTCATGAGACAATAACC	
174_PgAxT_PxT_IVA1_fw	CTCCCGGGACCTCAGAGTC	Cloning of pDae036
175_PgAxT_TadA8_IVA1_fw	GTAGCGGCTCTGGTTCCGGCTCTGGTAGCGGATCCAGTGAAGTTGAATTCAGCCATG	
176_PgAxT_TadA8_IVA1_rv	GGACTCTGAGGTCCCGGG	
177_AgPxT_TadA8_IVA2_rv	CCGGAACCAGAGCCGCTACCAGAGCCGGAACCATTAATGCTGCTCTGTGCTTTCT	Cloning of pDae037
182_dnfi_KanR_fw	ATGGATCTCGCGTCATTACGCGCTCAACAAATCGAACTGGCTTGATCCTTTGATCTTTTCTACGGGGTC	Amplification of kanamycin resistance gene from pET28b for construction of nfi k/o strain
183_dnfi_KanR_rv	TTAGGGCTGATTTGCTGTATAGCGCACGAACGCCGGACGTTCCGATGGCACTTTTCGGGGAAATGTG	
184_ugi-F	CAAACCCTGGGCTCTGGTG	
185_Amp-R	CAGCATCTTTTACTTTCACCAGC	
186_T7RNAP_PstI_rv	ATACTGCAGTTACGCGAACGCGAAGTCC	Cloning of pDae014
192_ugi_IVA-F	CTCAGGAGGAATTCACCATGGCATATGACCAACCTTTCCGACATC	Cloning of pDae044
193_ugi_IVA-R	CCGGAACCAGAGCCGCTACCAGAGCCGGAACCTAGCATCTTGATCTTGTTCTCTCC	
194_ugi-J23100-R	CTAGGACTGAGCTAGCCGTCAAAGGATCCCCGGGCTGCAG	Cloning of pDae043
195_ugi-J23100-RBS-F	GTACAGTGCTAGCCTAGAGTCAGGAGGAGACCTGCGATGACCAACCTTTCC	
223-pHyo250-IVA-F	AGCACCACCACCACCACCACTG	Cloning of pGE158
224-pHyo250-IVA-R	CATGGTATATCTCCTTCTTAAAGTTAAACAAAA	
225-TEM1-IVA-F	CCTCTAGAAATAATTTTGTTTAACTTTAAGAAGGAGATATACCATGAGTATTCAACATTTCCGTGTCG	
226-TEM1-IVA-R	CAGTGGTGGTGGTGGTGGTGCTCTCGAGTTACCAATGCTTAATCAGTGAGG	

231-AxT-32aa-F	CTCTGGTTCCGGCTCTGGTAGCGGATCCAGCGGCAGCGAGACTCCC	Cloning of pHW003
232-AxT-32aa-R	CCGCTACCAGAGCCGGAACCATTAATGCTGCTCTGTGCTTTCTTTTGTG	
235-GS-32aa-F	CTCAGAGTCCGCCACACCCGAAAGTAGTGAAGTTGAATTCAGCCATG	Cloning of pHW004
236-GS-32aa-R	GTCCCGGGAGTCTCGCTGCCACTGGATCCGCTACCA	
239_PstI_pBAD_fw	ATAACTGCAGGCATGCAAGC	Cloning of pDae028
240-Ftet-IVA-F	CTGTCAGACCAAGTTTACTCAACTG	Cloning of pGE158
241-Ftet-IVA-R	GACATAAGTCCATCAGTTCAACGG	
242-TcR-IVA-F	GACTTCCGTTGAACTGATGGACTTATGTCGTAATTCTCATGTTTGACAGCTTATCATC	
243-TcR-IVA-R	CGCAGTTGAGTAAACTTGGTCTGACAGTGGAGTGGTGAATCCGTTAGC	
245-G8aa-F	CCGAAAGTAGTGAAGTTGAATTCAGCCATGAATATTG	Cloning of pHW002
246-G8aa-R	GTGTGGCGGACTCTGAAACGGCTGGAGACTTAGTGG	
247-X8aa-F	CTCTGGTAGCGGCTCTAACACGATTAACATCGCTAAGAACG	Cloning of pHW001
248-X8aa-R	CCGGAACCATTAATGCTGCTCTGTGCTTTCTTTTGTG	
254_pBAD-NdeI_rv	AATCATATGCCATGGTGAATTCCTC	Cloning of pDae028
255-TadA-A113-R	GCACCGCGCTTGCTATTAC	
314-F-seq-F2	CATTAGGAAGCAGCCCAGTAGTAG	sequencing primer for target gene
315-F-seq-R2	GAGACGAAAGGGCCCGTACG	
316-UGI-RBS-F	CAATAAATAAGGAGGATTTTTTATGACCAACCTTTCCGACATCATAGAG	Cloning of pDae069
318-UGI-RBS-R	CTAGTACTCAAACAGAGCGCGCTCTGTTAGGATCCCCGGGCTGCAG	
341_NdeI_tadA_fw	TTACATATGTTGTCTGAAGTCGAATTTAGCCAC	Cloning of pDae028
357-F-IVA-F	TAACTCGAGCACCACCACCACCACCACTGAG	Cloning of target plasmid
367_T7RNAP_taa_PstI_rv	TTATCTGCAGTTACGCGAACGCGAAGTCC	Cloning of pDae028
368_28b_th_rv	CATATGGCTGCCGCG	Cloning of pET28b-TadA-7.10-T7RNAP
369_28b_ov_tadA_fw	GGTGCCGCGCGGCAGCCATATGTTGTCTGAAGTCGAATTTAGCCACG	
370_tadA_XTEN_ov_rv	GTGGCGGACTCTGAGGTCCCGGGAGTCTCGCTGCCGCTATCCGTCGAGGATTGCG	
371_ov_XTEN_T7RNAP_fw	GACTCCCGGGACCTCAGAGTCCGCCACACCCGAAAGTAACACGATTAACATCGCTAAGAAC	
399-F-J23100-F	GTACAGTGCTAGCCCTCTAGAAATAATTTTGTTTAACTTTAAGAAGGAG	Cloning of pDae120
400-F-J23100-R	CTAGGACTGAGCTAGCCGTCAAAATTTCGCGGGATCGAGATC	
445-F-IVA-R2	CATATGGCTGCCGCGCGGCAC	Cloning of target plasmid
446-MBP-IVA-F	GTGCCGCGCGGCAGCCATATG	Cloning of pDae117
447-MBP-IVA-R	GTGGTGGTGGTGGTGGTGCTCGAGTTAAGTCTGCGCGTCTTTCAGGGC	
448-GFP-IVA-F	GTGCCGCGCGGCAGCCATATGGCTAGCAAAGGAGAAGAACTC	Cloning of pDae118
449-GFP-IVA-R	GTGGTGGTGGTGGTGGTGCTCGAGTTAtTTGTAGAGCTCATCCATGCC	
451-F-MBP-IVA-R	CTCGAGTTAAGTCTGCGCGTC	Cloning of pDae119
452-F-MBP-IVA-F	GAGATCCGGCTGCTAACAAAG	
453-F-GFP-IVA-F	GAAAGACGCGCAGACTTAACTCGAGCCTCTAGAAATAATTTTGTTTAACTTTAAGAAGGAG	
454-F-GFP-IVA-R	GGGCTTTGTTAGCAGCCGG	
473_pBAD_dugi_fw__w187	AAGCTTGGCTGTTTTGGC	Cloning of pDae014
493-GFP-IVA-F2	GCTCATGAGCCCGAAGTGG	Cloning of pDae123
494-GFP-IVA-R2	GCTTGTTTCGGCGTGGGTATG	
495-KanR-IVA-F2	CATACCCACGCCGAAACAAGCCGAAAACTCACGTTAAGGGATTTTG	
496-KanR-IVA-R2	GCCACTTCGGGCTCATGAGCTGGCACTTTTCGGGGAAATGTG	
497-KO-K-GFP-F2	GGAAGGCCAGACGCGAATTATTTTTGATGGCGTTAACTCGCGAAAACTCACGTTAAGGGATTTTG	Amplification of kanamycin resistance gene and *gfp* gene from pDae123 for construction of lacZ k/o strain
498-KO-K-GFP-R2	CTTCAATCAGCGTGCCGTCGGCGGTGTGCAGTTCAACCACATCCGGATATAGTTCCTCCTTTCAG	
512_lacI_mid_forNGS_fw	GATATTTATGCCAGCCAGCC	For high-throughput sequencing
513_sopB_end_forNGS_rv	GACAGGTCTCGTGTTCCAAC	

Reference

1. Eom, G.E., Lee, H. and Kim, S. (2022) Development of a genome-targeting mutator for the adaptive evolution of microbial cells. *Nucleic Acids Res*, **50**, e38.
2. Park, H. and Kim, S. (2021) Gene-specific mutagenesis enables rapid continuous evolution of enzymes in vivo. *Nucleic Acids Res*, **49**, e32.
3. Melancon, C.E., 3rd and Schultz, P.G. (2009) One plasmid selection system for the rapid evolution of aminoacyl-tRNA synthetases. *Bioorg Med Chem Lett*, **19**, 3845-3847.
4. Gaudelli, N.M., Komor, A.C., Rees, H.A., Packer, M.S., Badran, A.H., Bryson, D.I. and Liu, D.R. (2017) Programmable base editing of A*T to G*C in genomic DNA without DNA cleavage. *Nature*, **551**, 464-471.
5. Badran, A.H. and Liu, D.R. (2015) Development of potent in vivo mutagenesis plasmids with broad mutational spectra. *Nat Commun*, **6**, 8425.
6. Richter, M.F., Zhao, K.T., Eton, E., Lapinaite, A., Newby, G.A., Thuronyi, B.W., Wilson, C., Koblan, L.W., Zeng, J., Bauer, D.E. *et al.* (2020) Phage-assisted evolution of an adenine base editor with improved Cas domain compatibility and activity. *Nat Biotechnol*, **38**, 883-891.
